# Supplementary material for: Multifunctional Neural Probes Enable Bidirectional Electrical, Optical, and Chemical Recording and Stimulation In Vivo
Source: Adv Mater. 2024 Nov 6;37(49):2408154. doi: 10.1002/adma.202408154 (PMC12053509; doi:10.1002/adma.202408154)
Supplement: Supplementary file 1 — Supporting Information [file ADMA-37-2408154-s001.pdf]

ADVANCED MATERIALS

Supporting Information

for *Adv. Mater.*, DOI 10.1002/adma.202408154

Multifunctional Neural Probes Enable Bidirectional Electrical, Optical, and Chemical Recording and Stimulation In Vivo

*Nicolette Driscoll, Marc-Joseph Antonini, Taylor M. Cannon, Pema Maretich, Greatness Olaitan, Valerie Doan Phi Van, Keisuke Nagao, Atharva Sahasrabudhe, Emmanuel Vargas Paniagua, Ethan J. Frey, Ye Ji Kim, Sydney Hunt, Melissa Hummel, Sanju Mupparaju, Alan Jasanoff, B. Jill Venton and Polina Anikeeva**

Supporting Information

Multifunctional Neural Probes Enable Bidirectional Electrical, Optical, and Chemical Recording and Stimulation In Vivo

*Nicolette Driscoll[#], Marc-Joseph Antonini[#], Taylor M. Cannon[#], Pema Maretich[#], Greatness Olaitan, Valerie Doan Phi Van, Keisuke Nagao, Atharva Sahasrabudhe, Emmanuel Vargas, Ethan Frey, Ye Ji Kim, Sydney Hunt, Melissa Hummel, Sanju Mupparaju, and Alan Jasanoff, Jill Venton, and Polina Anikeeva**

Supplementary Note 1. Notch Fiber.

During the design and fabrication of the POLI fiber, we fabricated a functionally equivalent fiber from the same components, termed the Notch fiber, using post-draw integration of carbon nanotube (CNT) yarn rather than convergence during the fiber thermal drawing process. This was done initially to test the concept in a cost-effective way, since convergence integration of six CNT microwires into the final POLI fiber required >60 m of costly 20 µm-diameter CNT microwire. Fabrication of the notch fiber was nearly identical to that of the final POLI fiber, including the same materials for the optical waveguide, poly(methyl methacrylate) (PMMA) core and THVP (terpolymer of tetrafluoroethylene, hexafluoropropylene, and vinylidene fluoride) cladding, with the same dimensions, however notched grooves along three sides of the preform were drilled in place of the six hollow channels found in the POLI fiber preform. These notches were filled with SEBS elastomer (styrene ethylene butylene styrene) to retain their shape during the drawing process. After the notch fiber was drawn, these strips of SEBS (final width of ~20 µm) were peeled from the notches on the sides of fiber sections, and the CNT microwire was pressed into these three groves along the sides of the fiber. The fiber was then coated with 5 µm of Parylene-C via chemical vapor deposition to electrically insulate and fix the CNT microwires in place. This produced a final fiber device that was functionally equivalent to the POLI fiber, but with three CNT electrodes rather than six.

Supporting Table S1. Refractive index and glass transition temperature of fiber constituents.

Material	Refractive index, *n* (wavelength)	Glass transition temperature T_g (°C)
PMMA	1.4956 (488 nm) [1]	105-120 [2]
PC	1.5976 (488 nm) [1]	130 - 170 [3]
COC	1.543 (488 nm) [4]	158 [5]
THVP	1.35 (589 nm) [6]	130 (Tm) [6]
SEBS	N/A	90 [7]

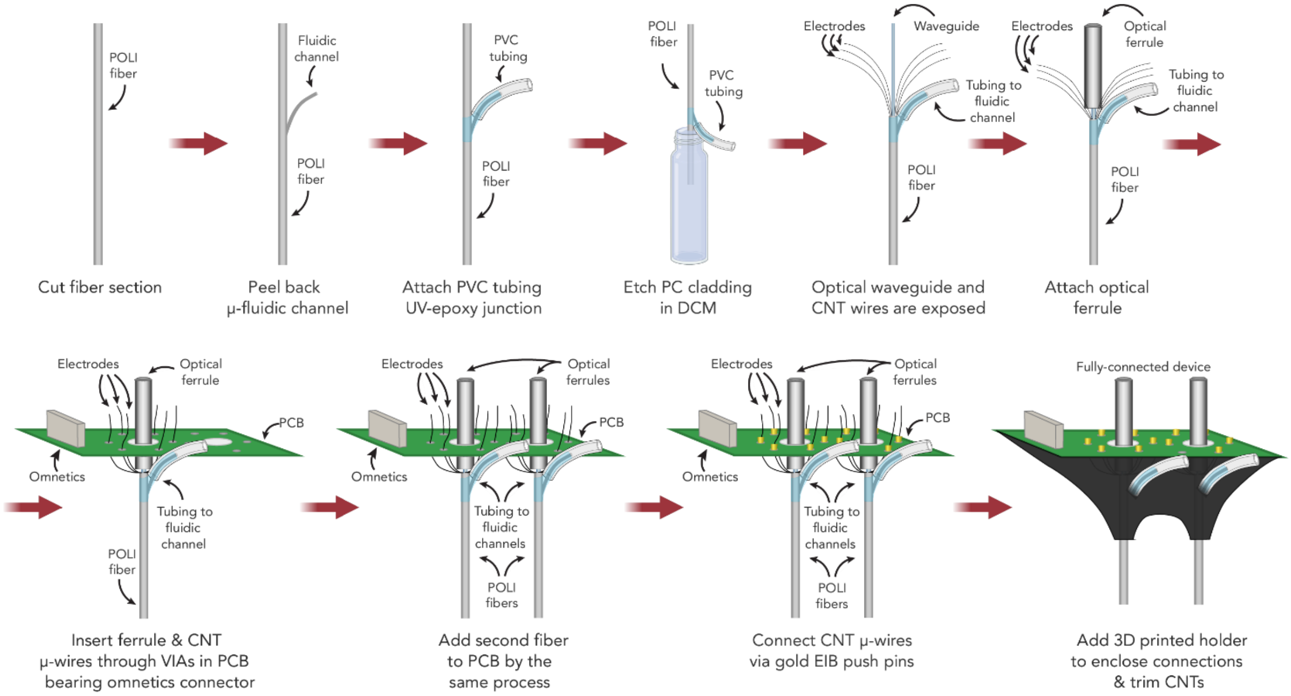

Supporting Figure S1. Diagram for POLI fiber interfacing with the multifunctional backend.

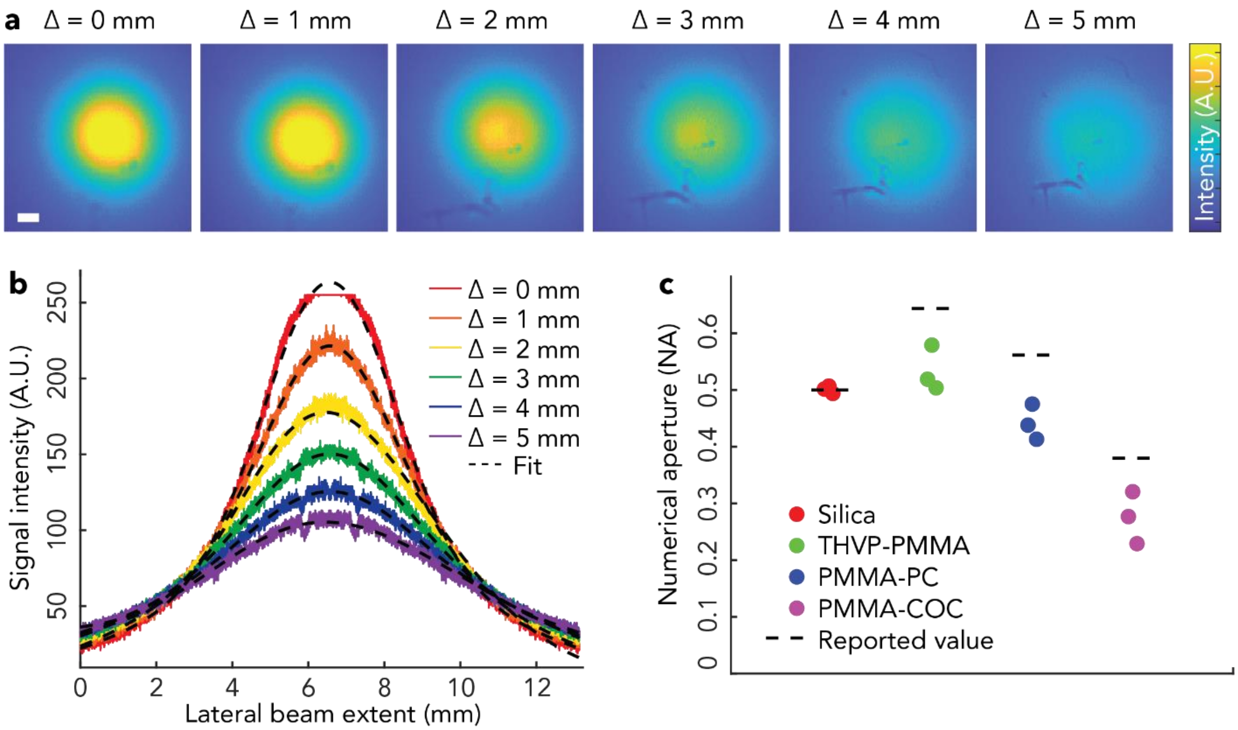

Supporting Figure S2. Quantification of numerical aperture (NA). (a) Representative beam profile images of PMMA-PC waveguide output light distribution at increasing distances (Δ) between the fiber tip and the camera sensor (scale bar = 1 mm). **(b)** Fitted gaussian beam profiles for PMMA-PC waveguide at each Δ. **(c)** NA quantification of each polymer waveguide (n = 3 fibers) based on beam dispersion method and comparison to values calculated from reported refractive indices (THVP-PMMA, PMMA-PC, PMMA-COC) or reported by manufacturer (silica). PMMA: poly(methyl methacrylate), PC: polycarbonate, COC: cyclic-olefin-copolymer, THVP: terpolymer of tetrafluoroethylene, hexafluoropropylene, and vinylidene fluoride, SEBS: styrene-ethylene-butylene-styrene.

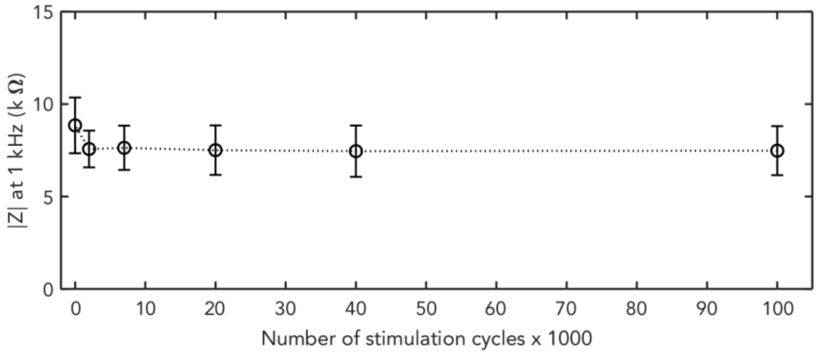

Supporting Figure S3. Stability of POLI fiber-integrated CNT electrodes for electrical stimulation. Mean impedance magnitude at the 1 kHz reference frequency of 20 µm CNT electrodes subjected to 100,000 cycles of stimulation pulsing with the following parameters: 200 µA, 50 Hz, biphasic, cathodic-first, 200 µs phases with 25 µs interphase interval (n = 5 electrodes, 100,000 pulses). A slight decrease in impedance is observed after the first 2000 cycles of stimulation due to the expected electrode conditioning effect, beyond which the impedance is stable.

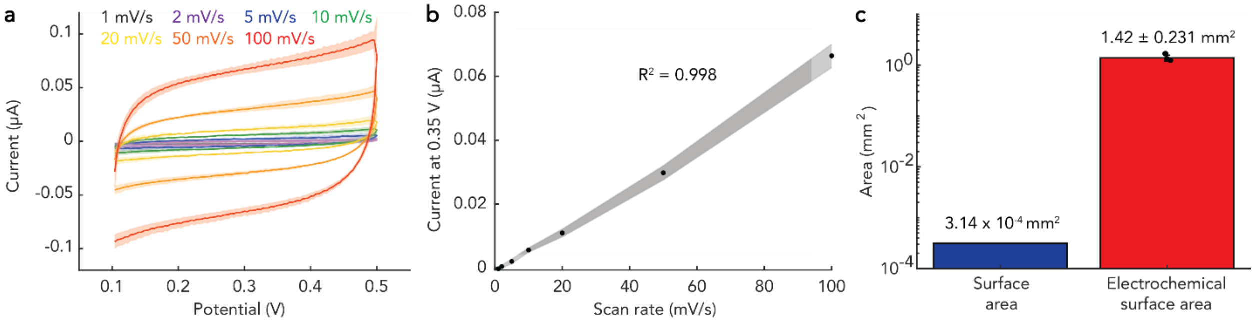

Supporting Figure S4. Electrochemical surface area (ECSA) characterization of CNT electrodes. (a) Cyclic voltammograms (CVs) were recorded in nitrogen-purged phosphate buffered saline (PBS) at progressively increasing scan rates over a voltage range displaying a flat current response. (n= 3 CNT electrodes) **(b)** Current values at the midpoint voltage (0.3 V) increase linearly with scan rate. Lines and shaded areas represent mean and standard deviation, respectively. **(c)** The slope of **(b)** was used to determine the ECSA of individual CNT electrodes (red) based on a specific capacitance estimated from literature (40 μF/cm^2)[8,9] and compared to the planar surface area for a 20 μm electrode (blue). Markers represent individual data points, and vertical lines denote standard deviation.

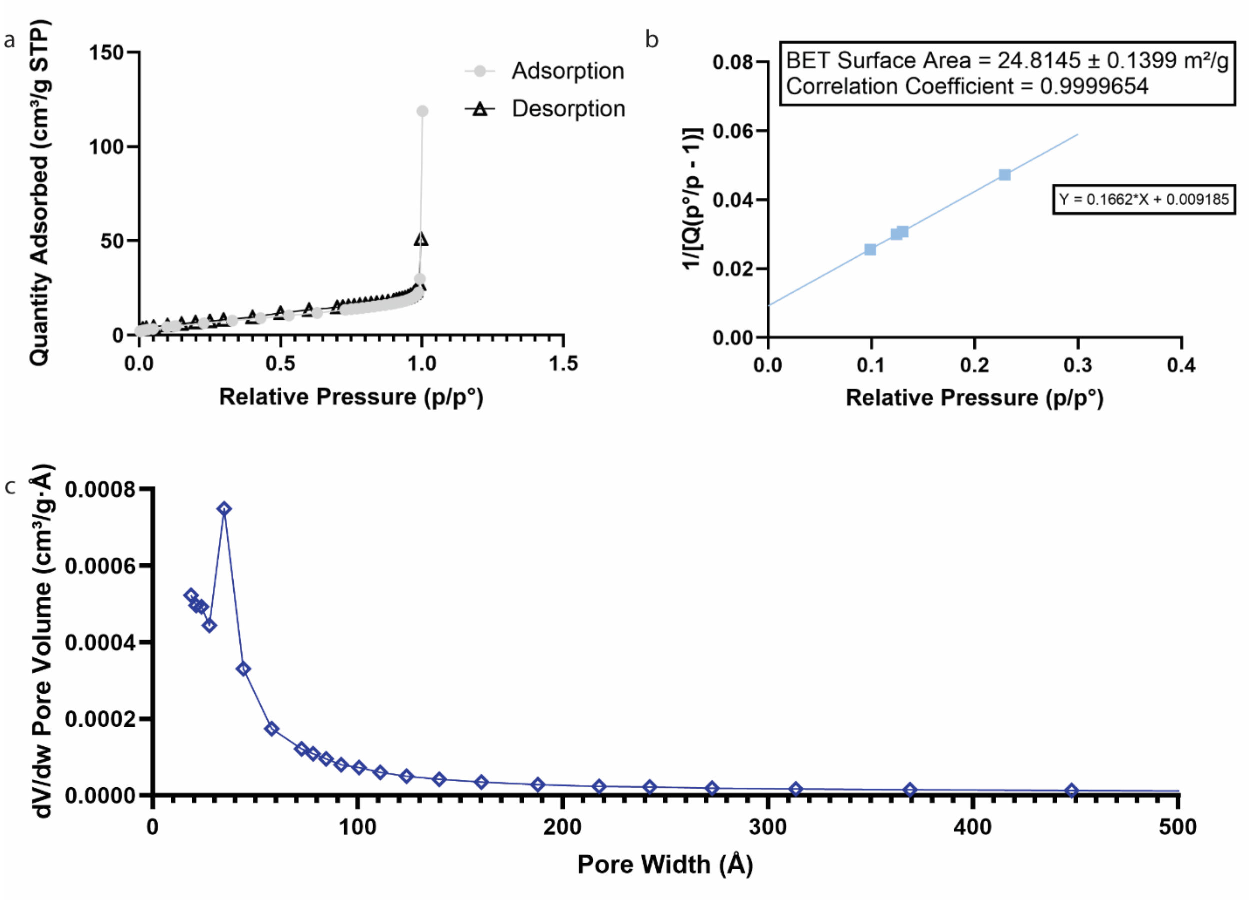

Supporting Figure S5. Brunauer, Emmett, and Teller (BET) surface area and porosity characterization of CNT electrodes. (a) The volume of nitrogen gas adsorbed upon a sample of 10 filament high strength 15 µm CNT fiber was measured using a Micromeritics ASAP 2020 Accelerated Surface Area and Porosimetry System as a function of relative pressure (equilibrium pressure (p)/ saturation vapor pressure (p^0)). (n = 1 CNT fiber segment) **(b)** The monolayer adsorbed gas quantity is then plotted against relative pressure. The slope and y intercept were extracted to determine the specific surface area of the sample (24.8145 ± 0.1399 m²/g). **(c)** Pore volume calculations were performed using the Barrett, Joyner, and Halenda (BJH) method based on a desorption model; the derivative of the cumulative desorbed volume (dV) was taken with respect to the effective pore width (dW) and plotted against the pore width indicating the prevalence varying pore sizes within the CNT.

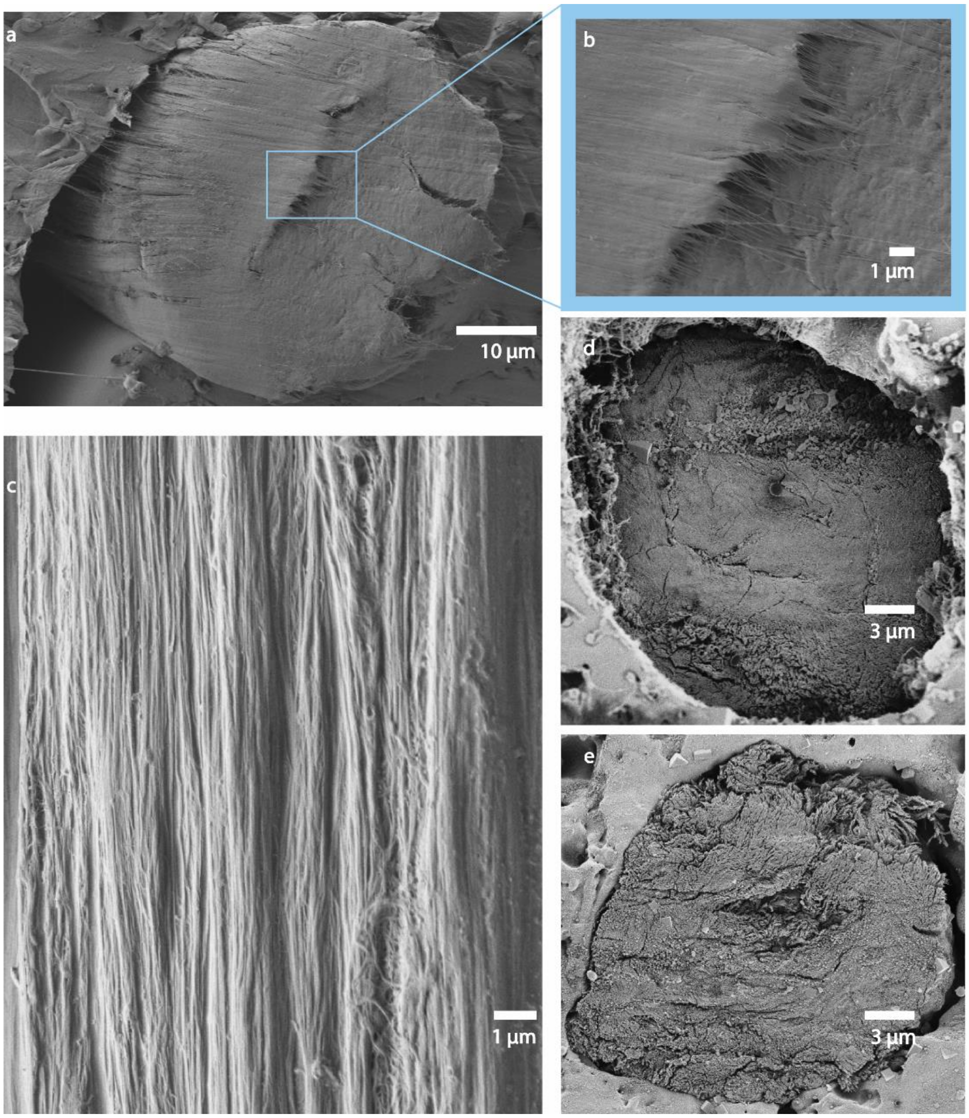

Supporting Figure S6. Scanning electron microscopy images of CNT fibers cut with a tungsten blade or an ultraviolet laser. **(a)** Cross-section of a CNT electrode converged within a POLI fiber cut with a tungsten blade; **(b)** A close-up image of CNT morphology at a fiber tip; **(c)** Lateral morphology of an as-purchased CNT fiber prior to thermal drawing; **(d)** CNT electrode within a UV laser-cut POLI fiber under the surface of the planar cross-section; **(e)** CNT electrode within a UV laser-cut POLI fiber cross-section in line with the surface of the planar cross-section.

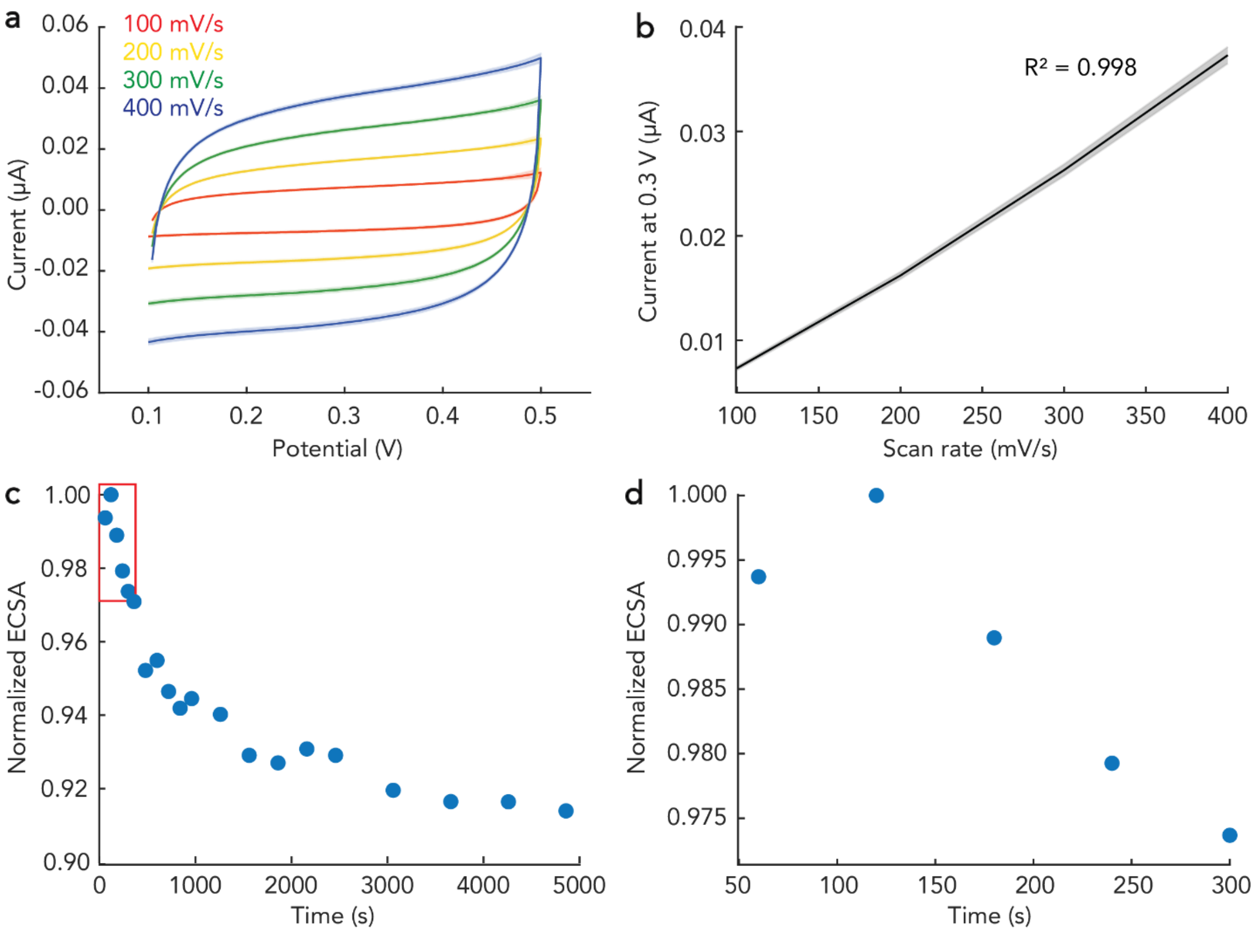

Supporting Figure S7. Effects of scan rate and time in PBS on CNT electrode ECSA. **(a)** CVs were recorded in nitrogen-purged PBS at increasing scan rates over a voltage range displaying a flat current response (n = 1 CNT electrode measured at 20 consecutive time points). **(b)** Current values measured at the midpoint voltage (0.3 V) increased linearly with scan rate. **(c), (d)** The slope of **(b)** was used to determine the ECSA of individual CNT electrodes based on a specific capacitance estimated from literature (40 μF/cm^2) at each time point. Prior to stabilization, a slight increase in ECSA was observed (**d**, red inset from **c**).

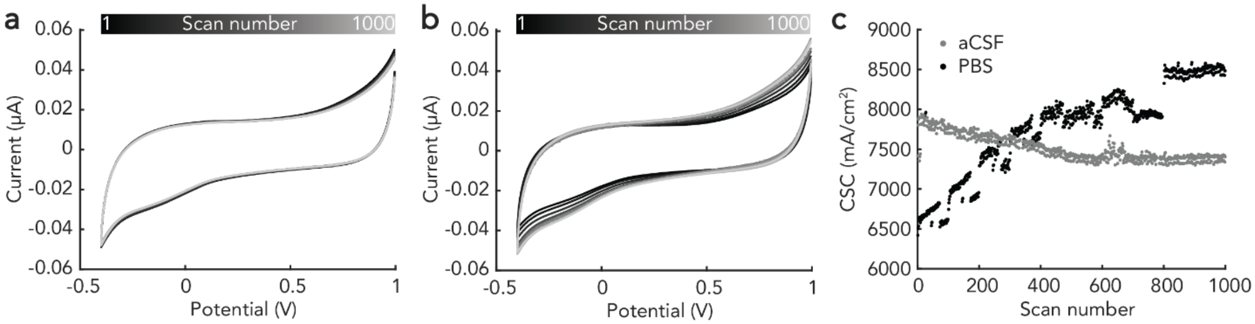

Supporting Figure S8. Cyclic voltammetry across 1000 cycles. CVs were acquired for CNT electrodes submerged in either artificial cerebrospinal fluid (aCSF, **a**, 1 electrode) or PBS (**b**, 1 electrode). **(c)** Charge storage capacity (CSC) was calculated at each time point, stabilizing at ~800 scans.

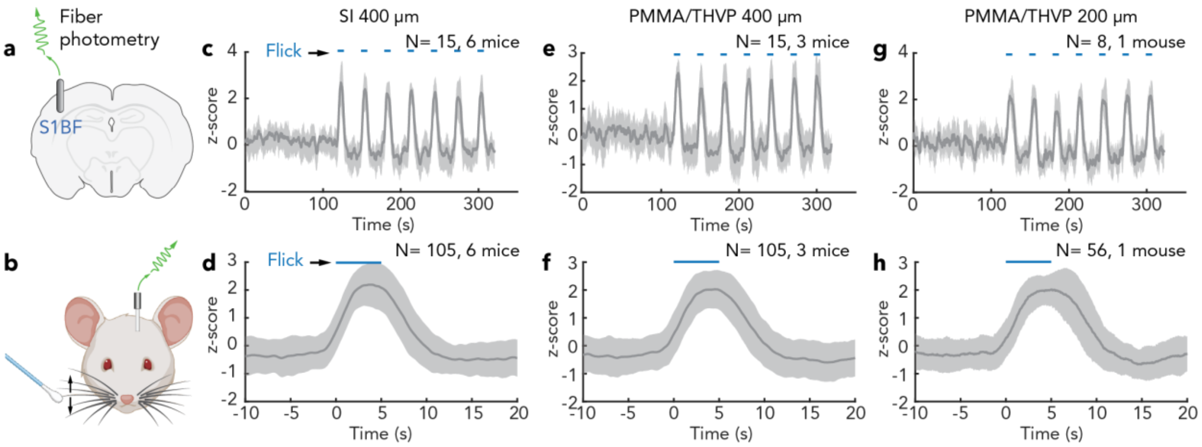

Supporting Figure S9. Validation of fiber photometry in vivo. **(a)** Schematic of the implantation site. Eight-week old Thy1-GCaMP6s mice were implanted with a 400 µm silica waveguide (n = 15 trials, 6 mice - 3 females and 3 males), 400 µm PMMA/THVP waveguide (n = 15 trials, 3 mice - 2 male and 1 female), or 200 µm PMMA/THVP waveguide (n = 8 trials, 1 female mouse). All implants targeted S1BF area. **(b)** Schematic of the experiment. The whiskers contralateral to the implantation site were mechanically stimulated with a brush, while neuronal activity was recorded via GCaMP6s photometry. **(c-h)** Average GCaMP6s signal recorded via a 400 µm silica waveguide (c-d), 400 µm PMMA/THVP waveguide (e-f), or a 200 µm (g,h) during the whisker stimulation experiment (c,e,g), or represented as a stimulation onset aligned response (d,f,h); each blue tick corresponds to a whisker brush. Data is represented as mean ± s.e.m.

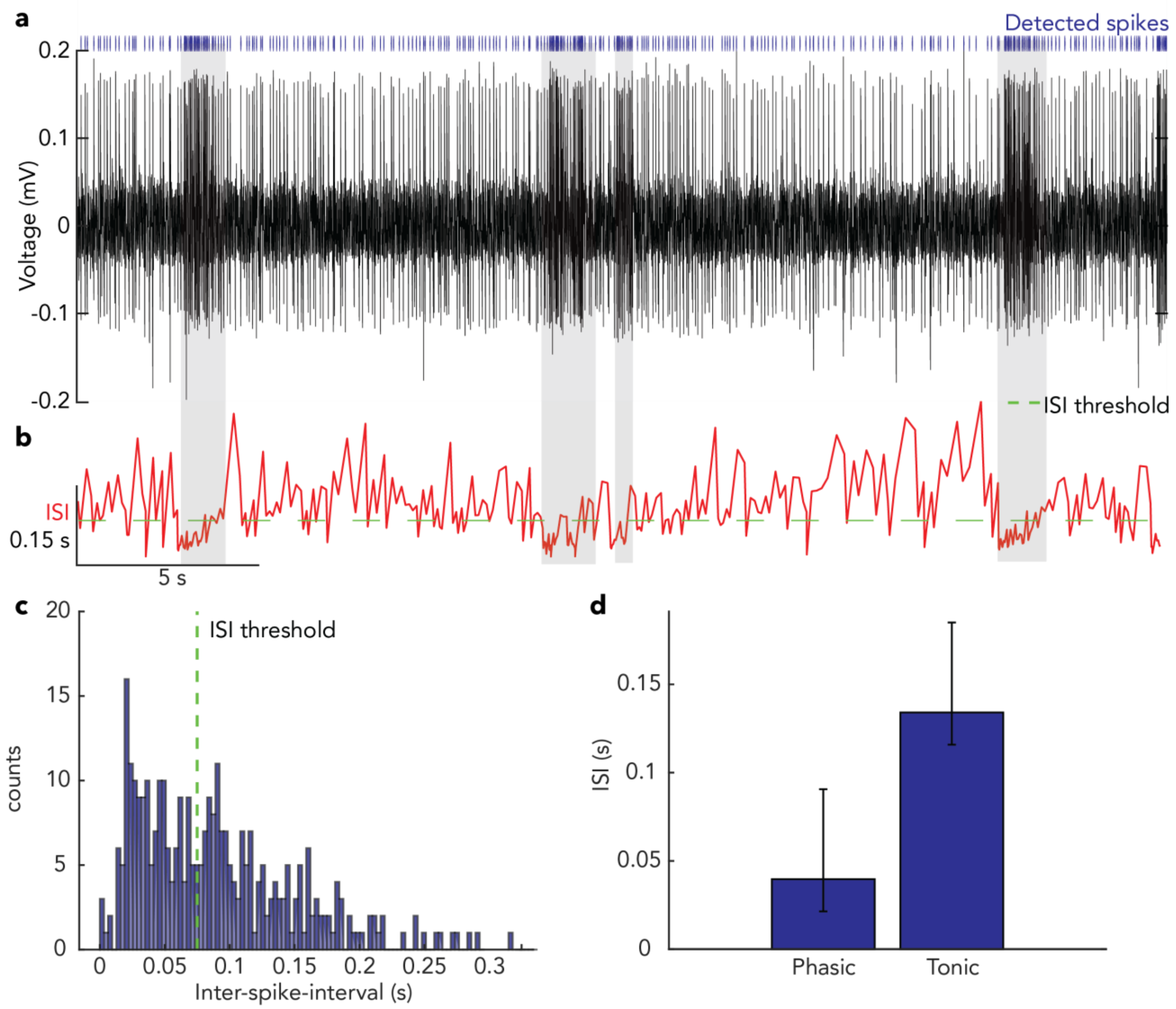

Supporting Figure S10. Quantification of endogenous spiking activity in the VTA. **(a)** Raw electrophysiological recording also shown in **Figure 4b**. **(b)** The inter-spike interval (ISI) for the electrophysiological data in (a) was quantified throughout periods of tonic and phasic electrophysiological activity. **(c)** An ISI threshold of 0.075 s (13 Hz) was chosen based on visual assessment to differentiate phasic and tonic activity (b). **(d)** Phasic activity below this threshold was found to have an average ISI of 0.040 s (25 Hz), and tonic activity above this threshold was found to have an average ISI of 0.13 s (7.5 Hz).

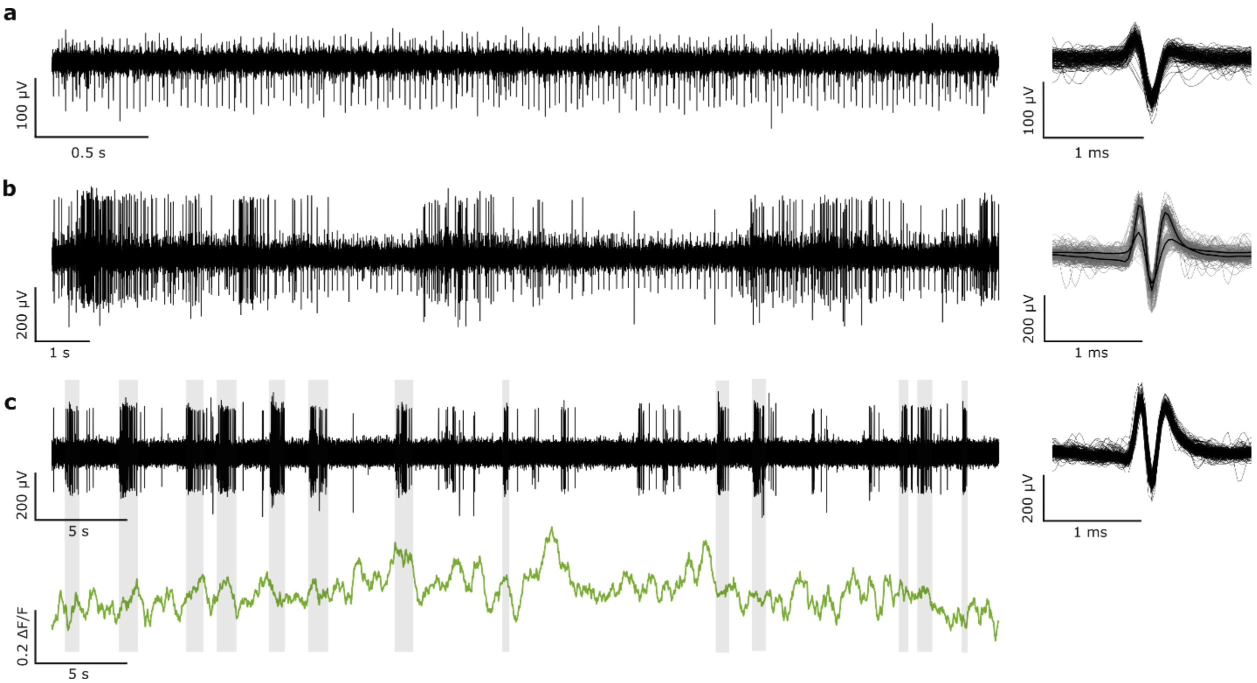

Supporting Figure S11. Endogenous firing of VTA neurons and corresponding DA transients recording in NAc. **(a)** Raw electrophysiological recording of endogenous firing activity in the VTA. Phasic firing was not observed in this animal. (n = 1 mouse) Extracted spike waveforms are shown to the right. **(b)** Raw electrophysiological recording of endogenous firing activity in the VTA of a different animal, this time with notable phasic activity. Two populations of sorted spike waveforms are shown to the right, with each mean waveform overlaid. (n = 1 mouse) **(c)** Time-aligned electrophysiological recording in the VTA with photometric recording of dLight1.1 fluorescent transients in the NAc of a third animal. (n = 1 mouse) Epochs of bursting are highlighted in grey. Extracted spike waveforms are shown to the right.

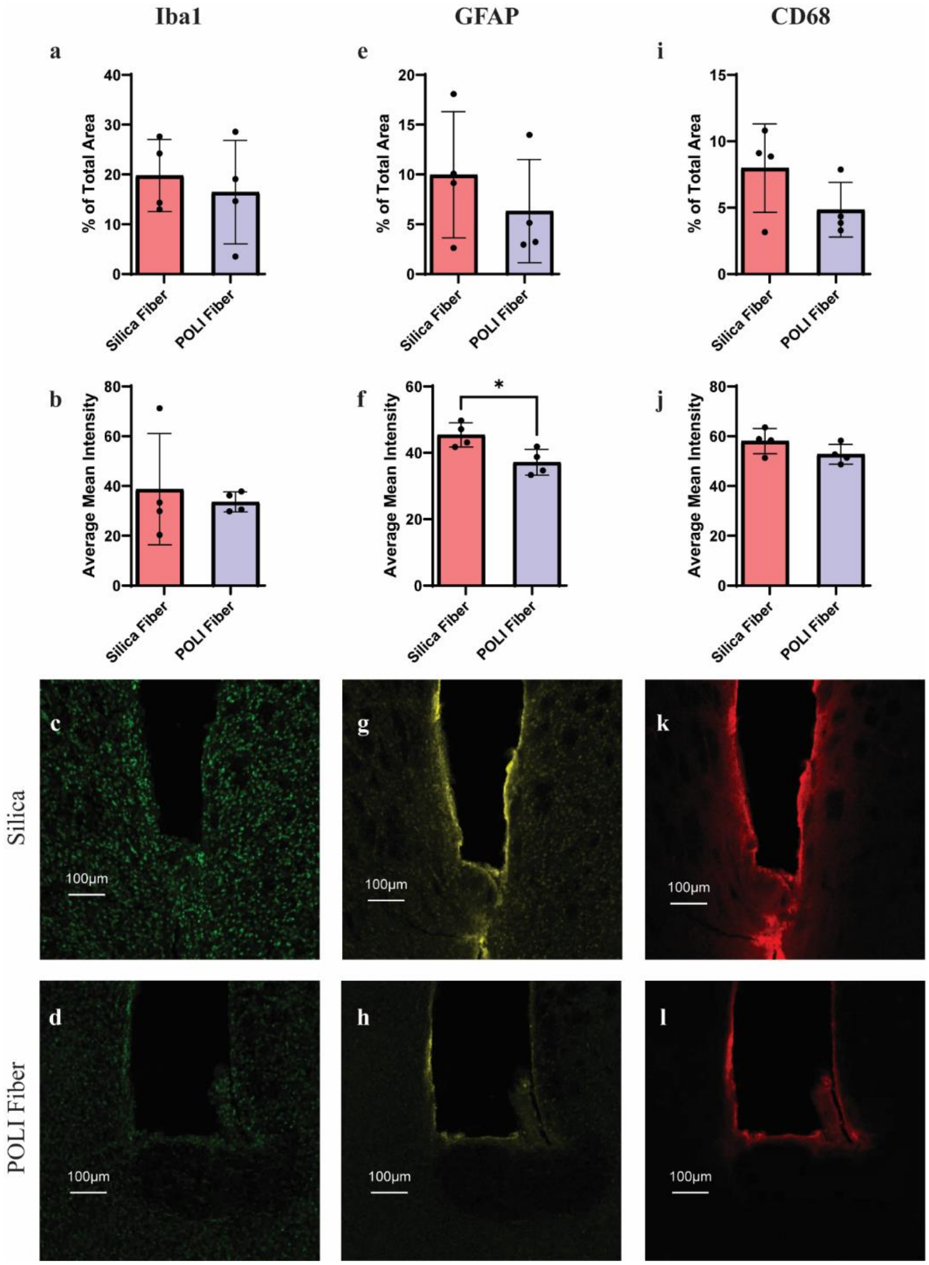

Supporting Figure S12. Immunohistochemical evaluation of chronically bilaterally implanted silica and POLI fibers. Immunofluorescent quantification of astrocytic (GFAP) and microglial (Iba1, CD68) markers surrounding a 300-400µm section of POLI-fiber and a 400 µm silica fiber implanted into the NAc of male $DAT^{IREScre}$ mice (n = 4, scale bar = 100 µm). Average fluorescent area and average fluorescence intensity of Iba1 **(a-d)**, GFAP **(e-h)**, and CD68 **(i-l)** as well as representative confocal images at the implant tips of POLI fiber (-1.25ML, +1.2AP, -4.3DV) and silica fiber (+1.25ML, +1.2AP, -4.3DV) 1 month post implantations. GFAP average mean intensity; p = 0.0421, Student's t-test. Data are presented as mean values +/- s.d.

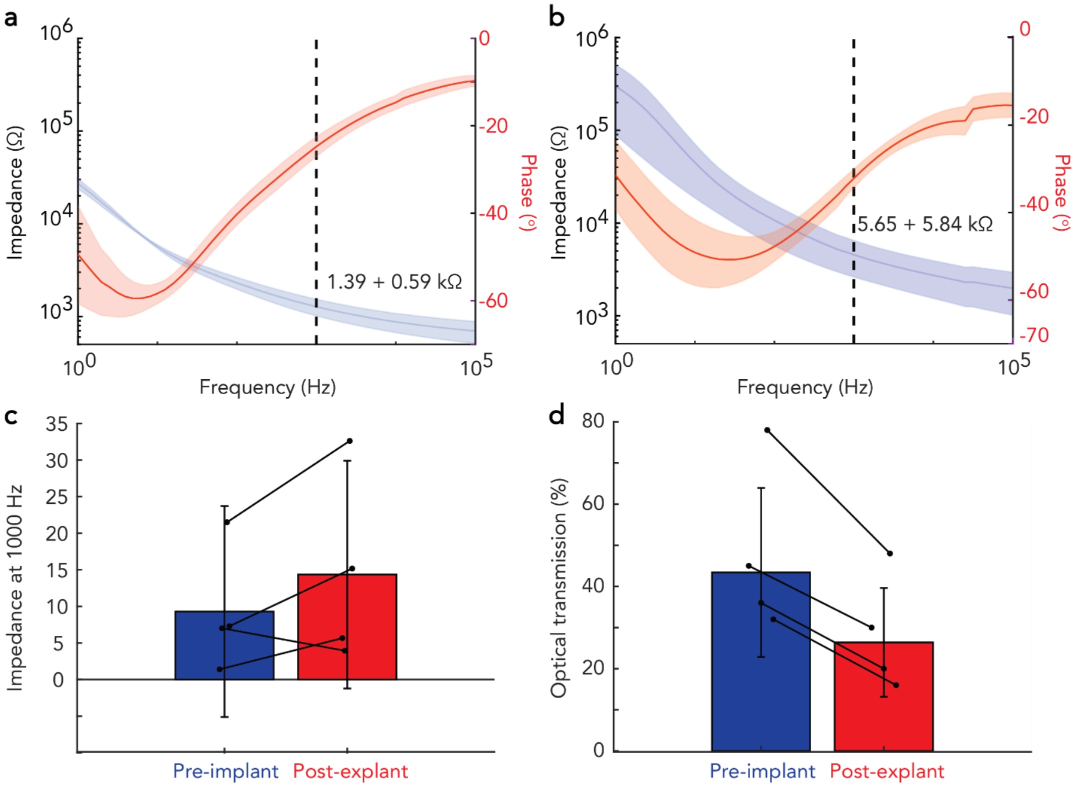

Supporting Figure S13. POLI fiber electrical and optical properties following explantation. **(a), (b)** Impedance (blue) and phase (red) spectra are shown for a representative fiber (n = 5 active electrodes) prior to implantation **(a)** and following explantation **(b)**. Lines and shaded areas mark mean and standard deviation. **(c), (d)** Impedance values at 1000 kHz **(c)** and optical transmission at 470 nm **(d)** are shown pre- (blue) and post-explantation (red). Markers in **(c)** indicate the mean value of n = 5 individual electrodes measured from each of n = 4 devices; markers in (d) indicate n = 4 individual waveguides. Vertical bars denote standard deviation.

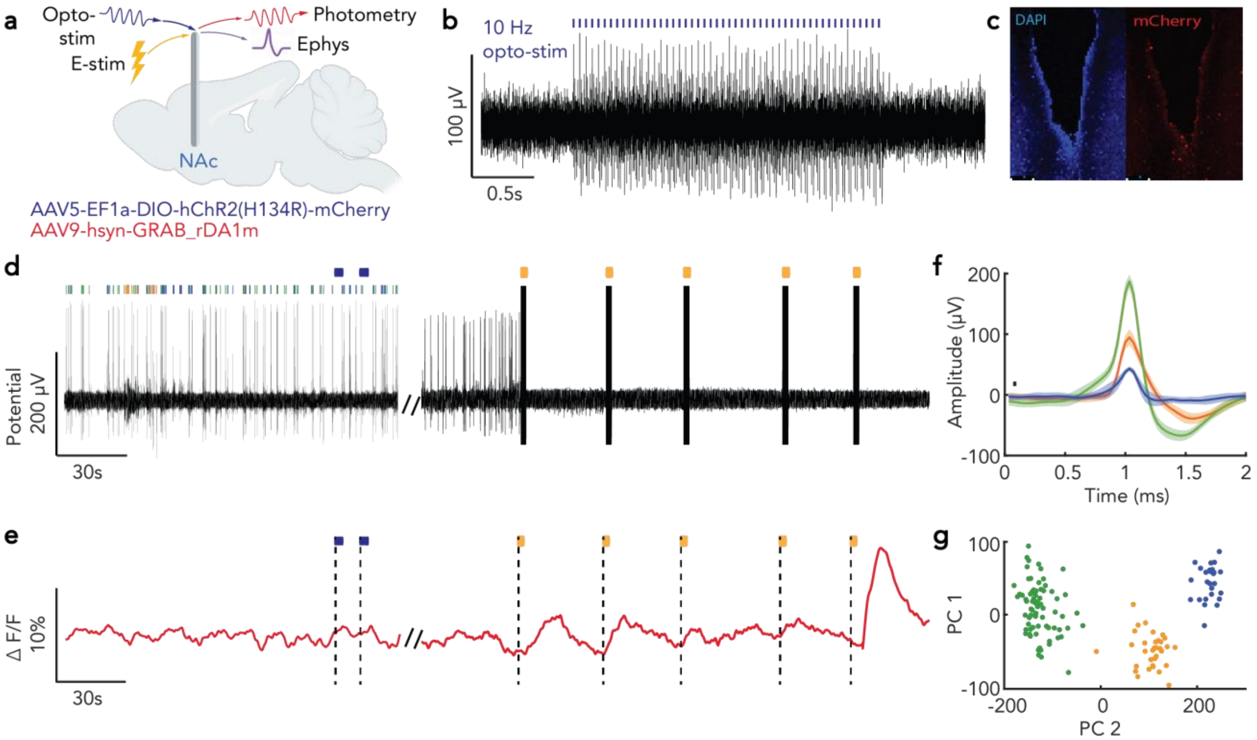

Supporting Figure S14. Single-site multimodal recording and stimulation. **(a)** POLI fibers were implanted in the NAc of DAT-Cre mice expressing ChR2-mCherry and $GRAB_{rDA1m}$ and used to record simultaneous electrophysiological and photometric (DA) responses to electrical and optical stimulation. (n = 4 mice, 2 male, 2 female) **(b)** Optical stimulation at 10 Hz evoked electrical activity in DAT neurons. **(c)** mCherry expression was confirmed at the tip of the fiber tract in the NAc using fluorescence microscopy (scale bar = 100 µm**)** **(d)** Electrical activity was recorded during optical stimulation (blue dashes) and electrical stimulation (yellow dashes), alongside DA release using photometry **(e)**. **(f)** Spikes detected as indicated in **(d)** were sorted into three populations **(g)** using principal component analysis. Hatch marks indicate a temporal break within the same continuous recording. Lines and shaded areas denote mean and standard deviation.

References

[1] I. D. Nikolov, C. D. Ivanov, *Appl. Opt., AO* **2000**, *39*, 2067.
[2] C. B. Roth, A. Pound, S. W. Kamp, C. A. Murray, J. R. Dutcher, *Eur. Phys. J. E* **2006**, *20*, 441.
[3] N. Shamim, Y. P. Koh, S. L. Simon, G. B. McKenna, *Journal of Polymer Science Part B: Polymer Physics* **2014**, *52*, 1462.
[4] A. Piruska, I. Nikcevic, S. H. Lee, C. Ahn, W. R. Heineman, P. A. Limbach, C. J. Seliskar, *Lab Chip* **2005**, *5*, 1348.
[5] J. A. Frank, M. J. Antonini, P. H. Chiang, A. Canales, D. B. Konrad, I. C. Garwood, G. Rajic, F. Koehler, Y. Fink, P. Anikeeva, *ACS Chemical Neuroscience* **2020**, *11*, 3802.
[6] "3M Dyneon™ THVP 2030GZ Fluorothermoplastic Granules," can be found under https://www.matweb.com/search/datasheet.aspx?matguid=d2a727ec073144fe8994ae1ea9c819bb&ckck=1, **n.d.**
[7] S. Zulfiqar, Z. Ahmad, M. Ishaq, S. Saeed, M. I. Sarwar, *J Mater Sci* **2007**, *42*, 93.
[8] H. Aldosari, A. Ali, M. A. Asghar, A. Haider, Y. Mehmood, Z. Iqbal, A. Nazir, M. Iqbal, *Journal of Science: Advanced Materials and Devices* **2023**, *8*, 100638.
[9] C. Mo, J. Jian, J. Li, Z. Fang, Z. Zhao, Z. Yuan, M. Yang, Y. Zhang, L. Dai, D. Yu, *Energy Environ. Sci.* **2018**, *11*, 3334.
